# A new double-antibody sandwich ELISA targeting *Plasmodium falciparum *aldolase to evaluate anti-malarial drug sensitivity

**DOI:** 10.1186/1475-2875-8-226

**Published:** 2009-10-12

**Authors:** Lucienne Tritten, Hugues Matile, Reto Brun, Sergio Wittlin

**Affiliations:** 1 Swiss Tropical Institute, Socinstrasse 57, CH-4002 Basel, Switzerland; 2 F. Hoffmann-La Roche Ltd., Grenzacherstrasse 124, CH-4070 Basel, Switzerland

## Abstract

**Background:**

The standard *in vitro *test to assess anti-malarial activity of chemical compounds is the [^3^H]hypoxanthine incorporation assay. It is a radioactivity-based method to measure DNA replication of *Plasmodium *in red blood cells. The method is highly reproducible, however, the handling of radioactive material is costly, hazardous and requires the availability of appropriate technology and trained staff. Several other ways to evaluate *in vitro *anti-malarial activity do exist, all with their own assets and limitations.

**Methods:**

The newly developed double-antibody sandwich ELISA described here is based on the properties of a non-overlapping pair of monoclonal antibodies directed against *Plasmodium falciparum *aldolase. This glycolytic enzyme possesses some unique nucleotide sequences compared to the human isoenzymes and has been highly conserved through evolution. Out of twenty possibilities, the most sensitive antibody pair was selected and used to quantitatively detect parasite aldolase in infected blood lysates.

**Results:**

A total of 34 compounds with anti-malarial activity were tested side-by-side by ELISA and the [^3^H]hypoxanthine incorporation assay. The novel ELISA provided IC_50_s closely paralleling those from the radioactivity-based assay (R = 0.99, p < 0.001). At the investigated assay conditions (72 h incubation time, parasitaemia = 0.3%), the assay was found to be reproducible and easy to perform.

**Conclusion:**

The newly developed ELISA presents several advantages over the comparative method, the [^3^H]hypoxanthine incorporation assay. The assay is highly reproducible, less hazardous (involves no radioactivity) and requires little and cheap technical equipment. Relatively unskilled personnel can conduct this user-friendly assay. All this makes it attractive to be employed in resource-poor laboratories.

## Background

Several techniques exist to measure anti-malarial activity of chemical compounds. The most commonly used method, especially in well-equipped laboratories, is the [^3^H]hypoxanthine incorporation assay [1]. This method is highly reproducible, however, the handling of radioactive material is costly, hazardous and quite complex and, therefore, problematic for resource-poor locations. Moreover, radioactive material is not uniformly authorized worldwide, limiting its application geographically. A low-cost alternative is the schizont maturation assay, standardized by the World Health Organization. However, this test can only be carried out by the experienced microscopist, is very labour-intensive and prone to individual variability. A method that is simple to establish, highly reproducible, that requires little technical equipment and could be applicable to a field laboratory, is the enzyme-linked immunosorbent assay (ELISA). A few commercialized ELISA tests are already available, targeting either *Plasmodium falciparum *lactate dehydrogenase (pLDH) or histidine-rich protein 2 (HRP2) [2-6]. Like *P. falciparum *aldolase, pLDH is very much conserved between *P. falciparum *isolates [7]. It presents some unique differences to the human LDH, and its level can be used to determine the drug susceptibility of malaria parasites [8]. The existing commercial kits testing pLDH are suitable with an initial parasitaemia of 0.005% of cultivated or natural strains [5]. HRP2 has recently been reported to show extensive protein sequence diversity (mainly insertions) in all of the analysed 75 *P. falciparum *isolates collected from geographically different areas [9]. Importantly, it was demonstrated that the HRP2 protein diversity had an effect on the sensitivities of the HRP2 detection antibodies. The same group also reports that the aldolase protein sequence shows no insertions by analysing 36 of the original 75 *P. falciparum *isolates [10]. This prompted us to develop a suitable double-antibody sandwich ELISA detecting *P. falciparum *aldolase to evaluate anti-malarial drug sensitivity. The newly developed aldolase ELISA was compared to the [^3^H]hypoxanthine incorporation assay, testing anti-malarial compounds such as OZ277 [11,12], artesunate (AS), chloroquine (CQ), pyrimethamine (PYR) and mefloquine (MEF).

## Methods

### Parasite cultivation

*Plasmodium falciparum *(NF54, Schiphol Airport, Netherlands) was cultivated in a modification of the medium previously described [12,13], consisting of RPMI 1640 supplemented with 0.5% ALBUMAX^® ^II, 25 mM HEPES, 25 mM NaHCO_3 _(pH 7.3), 0.36 mM hypoxanthine and 100 μg/ml neomycin. Human erythrocytes served as host cells. Cultures were maintained at 37°C in an atmosphere of 3% O_2_, 4% CO_2 _and 93% N_2 _in humidified modular chambers. The NF54 isolate was provided by F. Hoffmann-LaRoche Ltd (Basel, Switzerland).

### Chemicals and materials

OZ277 tosylate (MW: 565) was provided by J.L. Vennerstrom (Nebraska, USA), pyrimethamine (MW: 249) and mefloquine hydrochloride (MW: 415) were gifts from F. Hoffmann-LaRoche (Basel, Switzerland), artesunate (MW: 384) was donated by Guilin Pharma Corp. (Guilin Guangxi, China) and chloroquine diphosphate (MW: 516) was purchased from Sigma. Further anti-malarial compounds were obtained from the NGBS malaria programme, a consortium formed by the Novartis Institute for Tropical Diseases, the Genomics Institute of the Novartis Research Foundation, the Biomedical Primate Research Center and the Swiss Tropical Institute (compounds were provided by M. Rottmann, Swiss Tropical Institute Basel, Switzerland). All anti-malarial compounds were dissolved in dimethylsulfoxide (DMSO) at 10 mg/ml, except chloroquine, which was dissolved in water. The stock solutions were kept at 4°C for a maximum of six months. Dilutions were prepared from the stock solution in hypoxanthine-free culture medium immediately before use. [^3^H]hypoxanthine was purchased from Amersham Bioscience (Buckinghamshire, UK).

The recombinant *P. falciparum *aldolases P41/5 (S359T) and P41/7 (K365N) were obtained from lambdaGT11 phage expression libraries constructed with genomic DNA of the K1 isolate of *P. falciparum *and were kindly provided by H. Döbeli, (F. Hoffmann-La Roche Ltd, Basel, Switzerland) [14,15].

### Generation of hybridoma cell lines producing anti-*Plasmodium falciparum *aldolase antibodies

Anti-*P. falciparum *aldolase antibodies were identified by immunoprecipitation with Protein A-sepharose (GE Healthcare) in the soluble fraction of an infected culture. Hybridoma strains P.41-1/2-7, P.41-2/3-7, P.41-4/1-12, P.41-8/11-8 and P.41-24/10-11 [16] were grown in Iscove's modified Dulbecco's medium (Invitrogen) supplemented with 10%v/v FBS (Gibco), 1%v/v Penicillin-Streptomycin (Gibco), 1%v/v L-Glutamine (Gibco) in cell culture flasks, at 37°C, 5%CO_2_. Monoclonal antibodies (mAbs) were purified from hybridoma supernatant on ProteinG following the manufacturer's instructions (AKTAprimeTM, Amersham Biosciences, using HiTrapTM Protein G (GE Healthcare)). The detection antibody was coupled to horseradish peroxidase (HRP, SIGMA) as described by Nakane and Kawaoi [17]. The capture antibody was stored at 4°C in PBS supplemented with 0.02% NaN_3_. The HRP detection antibody was kept at 4°C in a PBS-50% glycerol solution supplemented with 10 mg/ml BSA.

### *In vitro *growth-inhibition assay

*Plasmodium falciparum *(NF54) growth was assessed by measuring the incorporation of the nucleic acid precursor [^3^H]hypoxanthine [18]. IC_50 _values were earlier found to be 0.91 ± 0.12 ng/ml for OZ277, 1.6 ± 0.1 for artesunate, 5.1 ± 0.8 for chloroquine, 5.8 ± 0.2 for mefloquine [11] and 5.6 ± 0.5 ng/ml for pyrimethamine [19]. Infected erythrocytes (100 μl per well with 2.5% haematocrit and 0.3% parasitaemia) were added to each drug titrated in 100 μl duplicates over a 64-fold range. After 48 h incubation, 0.5 μCi of [^3^H]hypoxanthine in 50 μl medium was added and plates were incubated for an additional 24 h. Parasites were harvested onto glass-fiber filters and radioactivity was counted using a Betaplate liquid scintillation counter (Wallac, Zurich). The results were recorded as counts per minute (cpm) per well at each drug concentration and expressed as a percentage of the untreated controls.

### Double antibody sandwich ELISA

96-well microtiter plates (Maxisorp, Nunc) were coated with 100 μl/well of the capture antibody P.41-24/10-11 (5 μg/ml in PBS) overnight at 4°C. The plates were then saturated with 200 μl/well of 1% BSA in PBS-Tween20 (0.05%) for 1 hour at 37°C. 50 μl of the test samples were then added and incubated 1 h at 37°C. Then, 50 μl/well of the detection antibody P.41-2/3-7 - HRP (0.8 μg/ml) were added, and the plates incubated 1 h in the dark at room temperature. Finally, 100 μl/well of the substrate (Tetramethylbenzidine) solution was added and the reaction stopped after 5 min with 100 μl 1 N H_2_SO_4_. The optical densities (ODs) were measured at 450 nm by an ELISA plate reader (Biotrak visible plate reader, Amersham Pharmacia Biotech). Between each incubation step, the plates were washed four times with PBS-Tween (0.05% Tween20).

### Statistics

Fifty percent inhibitory concentrations (IC_50_s) were determined in XLfit from IDBS by a 4 parameter logistic model. After controlling the normal distribution of the data, a correlation coefficient between the results obtained side-by-side by the two methods was determined by a standard Pearson's correlation analysis (Stata 9.1) and a Bland-Altman plot was performed, testing the agreement between them (Stata 9.1).

## Results

Five anti-recombinant aldolase monoclonal antibodies were tested both as capture as well as detection antibodies (HRP-labeled) in a double-antibody sandwich ELISA targeting *P. falciparum *recombinant aldolase P41/7 (highest concentration was 10 μg/ml). Ultimately, the antibody pair P.41-24/10-11; P.41-2/3-7+HRP was selected, showing a good specificity (steep curve) and the broadest dynamic range (OD span). When *P. falciparum *cultures (NF54) of e.g. initial parasitaemias of 0.3% or 0.03% were incubated for 72 hours and then lysed and analysed by ELISA, the observed ODs were 2.3 and 0.23, with a background signal (uninfected blood sample) of about 0.04. Having obtained such a solid signal to background ratio of > 50 at the higher starting parasitaemia (0.3%), and since 0.3% is the typical parasitaemia employed for the hypoxanthine assay, an ELISA with five anti-malarial compounds was performed under these conditions, side-by-side with a [^3^H]hypoxanthine incorporation assay. After 48 h, [^3^H]hypoxanthine dissolved in assay medium was added to the hypoxanthine incorporation assay, whereas the ELISA received the same volume of hypoxanthine-free assay medium. The geometric mean IC_50_s ± SDs obtained from the two methods are shown in Table 1, together with the expected [^3^H]hypoxanthine assay values.

**Table 1:** IC_50_s for OZ277, AS, CQ, PYR and MEF obtained from the aldolase ELISA and the [^3^H]hypoxanthine method with the *P. falciparum *strain NF54

**Anti-malarial compound**	**Drug assay *(n = 3)***	**IC 50 ± SD (ng/ml)**	**[^3^H]hypoxanthine data (ng/ml) from Vennerstrom *et al*, 2004; Maerki *et al*, 2006**
			

OZ277	[^3^H]hypoxanthine	0.36 (± 0.03)	0.91 (± 0.12)

OZ277	Aldolase ELISA	0.63 (± 0.18)	

			

AS	[^3^H]hypoxanthine	0.91(± 0.32)	1.6 (± 0.1)

AS	Aldolase ELISA	1.6 (± 0.3)	

			

CQ	[^3^H]hypoxanthine	3.8 (± 0.5)	5.1 (± 0.8)

CQ	Aldolase ELISA	5.3 (± 0.7)	

			

PYR	[^3^H]hypoxanthine	3.8 (± 0.6)	5.6 (± 0.5)

PYR	Aldolase ELISA	7.9 (± 2.1)	

			

MEF	[^3^H]hypoxanthine	4.3 (± 1.0)	5.8 (± 0.2)

MEF	Aldolase ELISA	6.9 (± 0.5)	

Geometric mean IC_50_s with 95% CIs for OZ277, artesunate (AS), chloroquine (CQ), pyrimethamine (PYR) and mefloquine (MEF) determined side-by-side by [^3^H]hypoxanthine assay and aldolase ELISA. Previously published IC_50_s for [^3^H]hypoxanthine assay are given for comparison. Mean values shown from n = 3 independent experiments.

**Figure 1:**
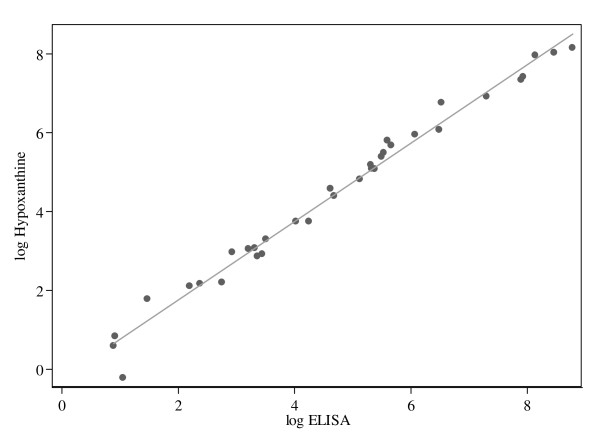
**Correlation of log IC_50_s obtained from the aldolase ELISA and the [^3^H]hypoxanthine method with the *P. falciparum *strain NF54**. 34 compounds were tested by ELISA and [^3^H]hypoxanthine incorporation (*n *= 1) with the *P. falciparum *strain NF54. The log IC_50 _in nM (or ng/ml for the standard compounds) from [^3^H]hypoxanthine incorporation is plotted against the log IC_50 _from ELISA, for each compound. (n = 34, correlation coefficient (R) = 0.991 and p-value < 0.001).

An excellent correlation coefficient of R = 0.991 (P < 0.001) was found when additional 29 anti-malarial compounds were tested and both log IC_50_s of all compounds, including OZ277, AS, CQ, PY and MEF, plotted (Figure 1).

**Figure 2:**
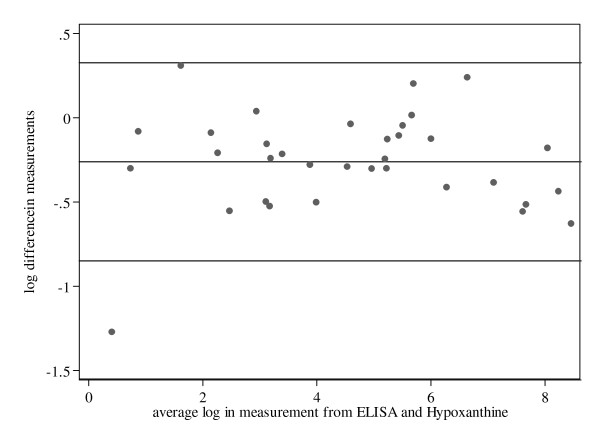
**Difference in log IC_50_s obtained from the the aldolase ELISA and the [^3^H]hypoxanthine method**. Bland-Altman plot of the difference in log IC_50_s in nM (or ng/ml for the standard compounds) for 34 anti-malarial compounds, determined by ELISA and [^3^H]hypoxanthine assay plotted against their mean. The mean difference is -0.261. The limits of agreement are represented by ± 2SD (-0.85 to 0.32).

To test the agreement between ELISA and [^3^H]hypoxanthine incorporation assay, the difference in log IC_50_s obtained from the two methods were plotted against their mean value in a Bland-Altman plot (Figure 2). The two methods were found to agree, as > 96% of the values lie in acceptable broad limits of agreement (-0.85 to 0.32).

## Discussion

The novel ELISA provides IC_50_s closely paralleling those from the standard [^3^H]hypoxanthine incorporation assay (Table 1), with P.41-24/10-11 as capture antibody and P.41-2/3-7-HRP as detection antibody targeting *P. falciparum *aldolase. The excellent correlation between IC_50_s determined by the [^3^H]hypoxanthine incorporation assay and by aldolase ELISA (R = 0.991, P < 0.001; Figure 1) was further confirmed by the Bland-Altman plot (Figure 2), showing a general trend of the two methods to agree nicely. The limits of agreement correlate with the findings of Noedl *et al *who compared the [^3^H]hypoxanthine incorporation assay and the HRP2 ELISA [20]. Interestingly, the aldolase ELISA tends in all cases to measure slightly higher IC_50_s than the [^3^H]hypoxanthine incorporation assay. However, this observation should have no impact on the power of the assay.

Aldolase is commonly used as a pan-malaria antigen in rapid diagnostic tests (RDTs) [21]. It can, therefore, not be ruled out that our monoclonal antibodies could cross-react with aldolase from other *Plasmodium *species. This should be kept in mind when embarking in future drug susceptibility testing efforts in species-overlapping zones.

Aldolase- and pLDH-based RDTs are reported to be less sensitive than HRP2-based tests, due to the transient presence in blood of the first [21,22]. However, since the lowest parasitaemia tested in this study (0.03%) did result in a solid signal to background ratio of ~5, the sensitivity is not expected to be an issue for drug sensitivity testing in the field. Furthermore, the aldolase amino acid sequence is highly conserved, preventing detection failures, as seen in the case of HRP2 [9]. The aldolase ELISA presents no drawbacks or assets over pLDH-based assays.

## Conclusion

The novel aldolase ELISA assay is highly reproducible, less hazardous than the [^3^H]hypoxanthine incorporation assay and requires little and cheap technical equipment. Relatively unskilled personnel can conduct this user-friendly assay. All this makes it attractive to be employed in resource-poor laboratories.

The fact that the *P. falciparum *aldolase has been highly conserved during evolution [10] renders aldolase an antibody target of choice for the analysis of field isolates. It also provides a considerable advantage over the HRP2 ELISA, since the HRP2 gene sequence is showing an extensive diversity, altering the sensitivity of some commercial diagnostic tests.

However, before proceeding to drug susceptibility testing in endemic countries, further investigations with more parasite isolates should be performed in order to determine the lowest measurable parasitaemia (sensitivity) of the aldolase ELISA as well as if the here described monoclonal antibodies cross-react with aldolase from other *Plasmodium *species.

## Competing interests

The authors declare that they have no competing interests.

## Authors' contributions

LT was the student in charge of developing the aldolase ELISA and did most of the laboratory work. HM, SW and RB supervised the project, directed the research and were involved in writing the manuscript. All authors have read and approved the final manuscript.
